# Cultural Continuity in a Reservation Nursing Home

**DOI:** 10.1093/geroni/igab046.897

**Published:** 2021-12-17

**Authors:** Pamela Monaghan-Geernaert

**Affiliations:** Northern State University, Aberdeen, South Dakota, United States

## Abstract

Throughout our lifespan we experience the culture of our families and communities. Our cultural selves guide our understanding of health and illness. However the health care system often ignores our culture in the delivery of care. This can have devastating effects on individuals and particularly the elderly. This presentation reviews a case study of a tribally owned and operated nursing home. The emphasis on maintaining cultural activities, feasting on traditional foods, offering sacred practices led to high satisfaction of the health care experience by residents and staff. Creating this environment was difficult and barriers in culturally responsive care delivery will also be discussed.

